# RAB-Like 2 Has an Essential Role in Male Fertility, Sperm Intra-Flagellar Transport, and Tail Assembly

**DOI:** 10.1371/journal.pgen.1002969

**Published:** 2012-10-04

**Authors:** Jennifer C. Y. Lo, Duangporn Jamsai, Anne E. O'Connor, Claire Borg, Brett J. Clark, James C. Whisstock, Mark C. Field, Vicki Adams, Tomomoto Ishikawa, R. John Aitken, Belinda Whittle, Christopher C. Goodnow, Christopher J. Ormandy, Moira K. O'Bryan

**Affiliations:** 1 Department of Anatomy and Developmental Biology, Monash University, Melbourne, Victoria, Australia; 2 Department of Biochemistry and Molecular Biology, Monash University, Melbourne, Victoria, Australia; 3 The Department of Pathology, University of Cambridge, Cambridge, United Kingdom; 4 Australian Phenomics Facility, John Curtin School of Medical Research, Australian National University, Canberra, ACT, Australia; 5 School of Environmental and Life Sciences, University of Newcastle, Callaghan, New South Wales, Australia; 6 The Garvan Institute of Medical Research, Darlinghurst, New South Wales, Australia; Washington University School of Medicine, United States of America

## Abstract

A significant percentage of young men are infertile and, for the majority, the underlying cause remains unknown. Male infertility is, however, frequently associated with defective sperm motility, wherein the sperm tail is a modified flagella/cilia. Conversely, a greater understanding of essential mechanisms involved in tail formation may offer contraceptive opportunities, or more broadly, therapeutic strategies for global cilia defects. Here we have identified Rab-like 2 (RABL2) as an essential requirement for sperm tail assembly and function. RABL2 is a member of a poorly characterized clade of the RAS GTPase superfamily. RABL2 is highly enriched within developing male germ cells, where it localizes to the mid-piece of the sperm tail. Lesser amounts of *Rabl2* mRNA were observed in other tissues containing motile cilia. Using a co-immunoprecipitation approach and RABL2 affinity columns followed by immunochemistry, we demonstrated that within developing haploid germ cells RABL2 interacts with intra-flagella transport (IFT) proteins and delivers a specific set of effector (cargo) proteins, including key members of the glycolytic pathway, to the sperm tail. RABL2 binding to effector proteins is regulated by GTP. Perturbed RABL2 function, as exemplified by the Mot mouse line that contains a mutation in a critical protein–protein interaction domain, results in male sterility characterized by reduced sperm output, and sperm with aberrant motility and short tails. Our data demonstrate a novel function for the RABL protein family, an essential role for RABL2 in male fertility and a previously uncharacterised mechanism for protein delivery to the flagellum.

## Author Summary

A greater understanding of the mechanism of male fertility is essential in order to address the medical needs of the 1 in 20 men of reproductive age who are infertile. Conversely, there remains a critical need for additional contraceptive options, including those that target male gametes. Towards the aim of filling these knowledge gaps, we have used random mutagenesis to produce the Mot mouse line and to identify RABL2 as an essential regulator of male fertility. Mice carrying a mutant *Rabl2* gene are sterile as a consequence of severely compromised sperm motility. Using biochemical approaches we have revealed that RABL2 binds to components of the intraflagellar transport machinery and have identified a number of RABL2 binding (effector) proteins. The presence of the Mot mutation in RABL2 leads to a significantly compromised ability to deliver binding proteins into the sperm tail. RABL2 is predominantly produced in male germ cells; however, lower levels are notably produced in organs that contain motile cilia (hair like structures involved in fluid/cell movement), thus raising the possibility that RABL2 may be involved in a broader set of human diseases collectively known as primary cilia dyskinesia.

## Introduction

Infertility affects at least 1 in 20 men of reproductive age [1] and for the majority, the underlying causal mechanism remains unknown. This, and the absence of effective male-based contraceptives, stems from a fundamental lack of knowledge of the genes and pathways required to form functional sperm.

Spermatozoa are produced within the seminiferous epithelium of the testis through a series of processes including stem cell renewal, meiosis and a radical differentiation process, termed spermiogenesis, wherein haploid germ cells are transformed into highly polarized cells with the potential for motility and fertilization. The mammalian sperm tail, like motile cilia and flagella from all species, contains an axoneme at its core composed of a 9+2 microtubule arrangement. The axoneme develops from a centriole/basal body at the base of the sperm head and functions to metabolize ATP and generate microtubule sliding and motility [2]. Unlike the majority of other cilia however, the sperm tail possesses peripherally arranged accessory structures including the fibrous sheath and outer dense fibers which impart directionality to tail beating, protection against shearing forces, and in the case of the fibrous sheath is a scaffold for enzymes involved in glycolysis and the generation of at least a proportion of the ATP required as fuel for axoneme movement [3]. The mechanisms by which the sperm tail is assembled remain almost completely unknown. Defects in sperm axoneme function result in asthenospermia (abnormal sperm motility) [4]. Global defects in motile axoneme function result in primary ciliary dyskinesia (PCD), a syndrome characterized by variable presentations of recurrent respiratory tract infections, male infertility, dextrocardia (Kartegener's syndrome) and hydrocephalus [5].

Using a random mutagenesis approach, we have identified RABL2 as being essential for sperm tail function and male fertility. RABL proteins are a poorly characterized sub-family of the Ras GTPase superfamily originally discovered in *Trypanosomes* and *Chlamydomonas* as an essential component of the intra-flagellar transport (IFT) particles required for primary cilia function [6], [7]. Here we have demonstrated that RABL2 is essential for sperm flagella, a motile cilia assembly. Biochemically, RABL2 function is regulated by GTP, it binds to components of the IFT complex B machinery and is involved in the delivery a set of cargo protein either to, or within, the developing flagellum.

## Results/Discussion

### RABL2 is required for male fertility

**Figure 1:**
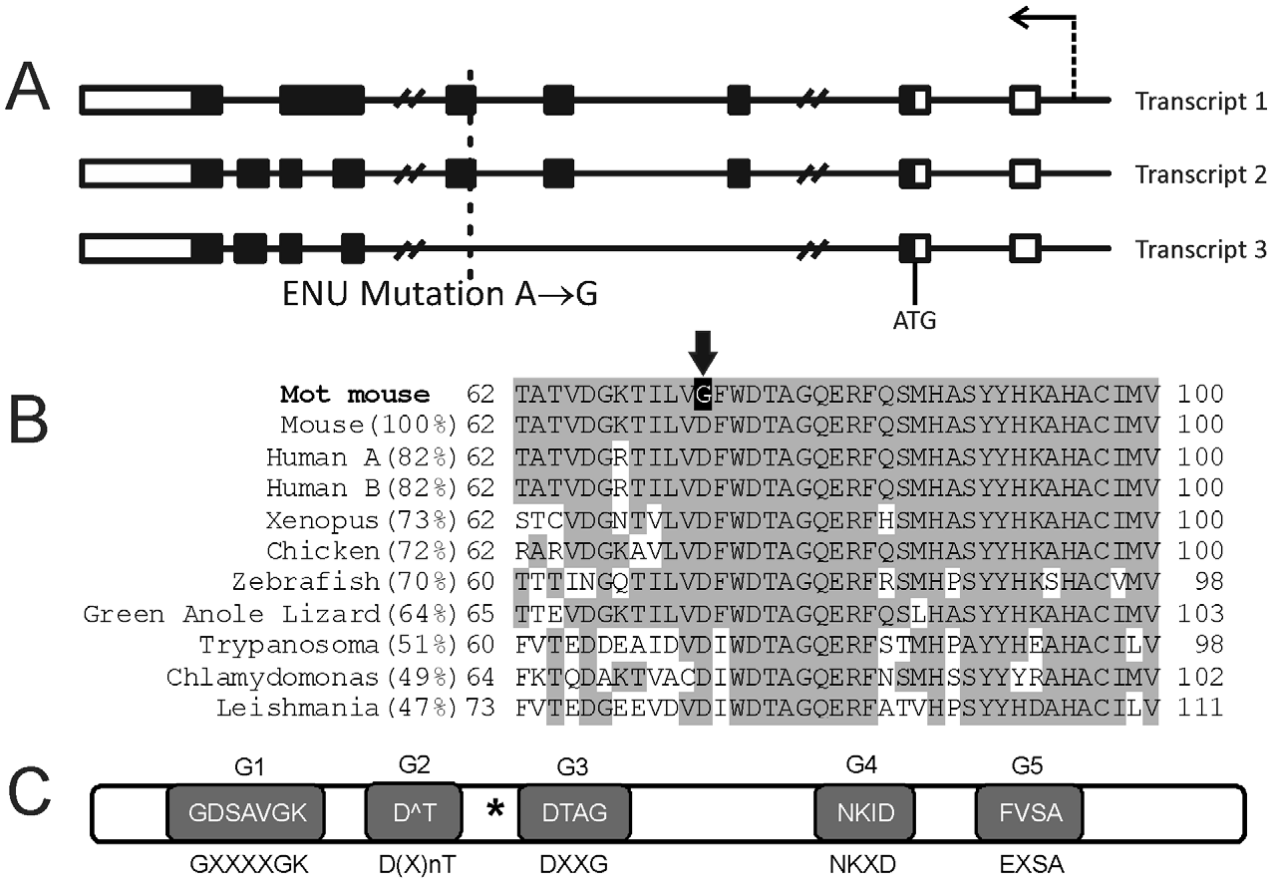
The Mot mouse line contains a point mutation in the *Rabl2* gene. (A) The Mot line contains a single A to G substitution in the first codon of exon 5 which would affect RABL2 isoforms (transcript) 1 and 2 but not the putative isoform 3. Boxes indicate exons (white are non-coding sequences and black are protein coding sequences), lines indicate introns. The arrow indicates the position of the promoter and the transcriptional direction. ATG indicates the translation initiation site. (B) The *Rabl2^Mot/Mot^* mutation results in the conversion of an evolutionarily conserved aspartic acid (D) into a glycine (G) (arrow). (C) RABL2 contains all five consensus motifs involved in GTP binding in RAB proteins. The consensus motif sequence is indicated below each shaded box and the actual RABL2 sequence indicated in the box. The asterisk indicates the position of the Mot mutation.

In an effort to identify genes critically involved in male fertility, we used N-ethyl-nitrosourea (ENU) to randomly mutate the mouse genome. Mice were mutated on a C57BL6 background then outbreed onto the CBA strain for two generations to facilitate mutation mapping, after which mice were maintained on a mixed background through inter-crossing. Mouse lines carrying mutations causing male sterility were identified using breeding trials [8], [9]. The Mot line presented with male sterility with a frequency of one in four individuals, and was thus strongly suggestive of a recessive mutation. Mapping narrowed the causal mutation to a region on chromosome 15 (bp 64,938,858 and 93,141,531) containing 49 genes. Of these genes, 32 were expressed within the testis as indicated in EST expression databases, and were thus potentially causal in the Mot phenotype. The protein-coding regions and intron-exon boundaries of all 32 genes were amplified and sequenced and a single homozygous A to G mutation was identified in exon 5 of the *Rabl2* gene in all affected males (*Rabl2Mot/Mot*) (Figure 1A). No other mutations were found within the linkage region. Unaffected (fertile) males possessed either homozygous wild type alleles (*Rabl2WT/WT*) or were heterozygous for the wild type and Mot allele (*Rabl2^WT/Mot^*). The Mot mutation resulted in the substitution of an aspartic acid (D, negatively charged) for a glycine (G, non-polar) at amino acid 73 of the predicted RABL2 isoforms 1 (ENSMUST00000058058) and 2 (ENSMUST00000023294), while the predicted isoform 3 (ENSMUST00000094056) would be unchanged as a consequence of exons 3–5 being removed by splicing (Figure 1A–1B). Thus, *Rabl2^Mot/Mot^* males were sterile because of the single amino acid substitution in isoforms 1 and 2 of RABL2.

Of note, the frequency and the 100% association between the D73G mutation and male sterility was unchanged following 7 generations of backcrossing onto a pure C57BL6 background adding further weight to the identity of the *Rabl2* mutation as causal of the phenotype (data not shown). As indicated results contained herein were generated using mixed background mice.

### Mot males are sterile as a consequence of abnormal haploid germ cell development and sperm immotility

**Figure 2:**
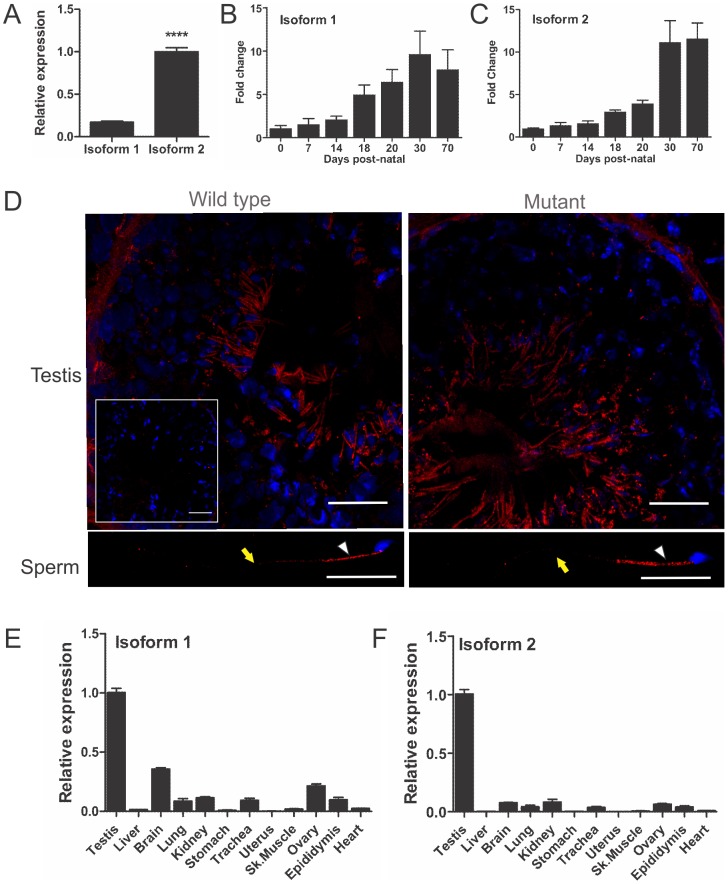
*Rabl2* expression and localization. (A) The adult mouse testis expresses mRNA for *Rabl2* isoform 1 and isoform 2, of which isoform 2 is more highly expressed. N = 3 mice per genotype (*** p<0.001). Relative *Rabl2* isoform 1 (B) and isoform 2 (C) expression in the developing post-natal testis indicates that both isoforms are predominantly produced within haploid germ cells which first appear at day 20 post-natal. The day zero value was set to 1 and all other ages expressed relative to this value. (D) RABL2 localization (red) within wild type and Mot homozygous mutant testis tissue and caudal epididymal sperm. The inset indicates staining obtained when the primary antibody was pre-absorped with excess immunizing peptide prior to immunofluorescence. The white arrows indicate the position of the mid-piece of the sperm tail. The yellow arrow indicates the position of the principal piece. DNA was labelled using DAPI (blue). The relative expression of *Rabl2* isoform 1 (E) and 2 (F) in adult tissues. N = 3 mice per group. The testis was set at 1 and all other tissues expressed relative to this value.

Quantitative PCR analysis on testes of different ages during post-natal development and the establishment of spermatogenesis revealed high levels of isoform 2 and lower levels of isoform 1 mRNAs expression (Figure 2A). Both isoforms 1 and 2 were most highly expressed from post-natal day 18 when haploid germ cells first appear in the germinal epithelium (Figure 2B–2C). Immunofluorescent labelling, using a monoclonal antibody generated against RABL2 isoform 2, confirmed RABL2 localized predominantly to the haploid compartment of the testis (Figure 2D). Within epididymal sperm, RABL2 was localized to the mid-piece of the tail (Figure 2D). Pre-absorption of the antibody with the immunizing peptide prior to immunofluorescent labelling resulted in the elimination of staining (Figure 2D inset), thus supporting the specificity of immunolablelling. Immunfluorescent labelling of testis sections from *Rabl2^Mot/Mot^* animals resulted in a similar localization of RABL2 as that observed in wild type samples (Figure 2D).

**Figure 3:**
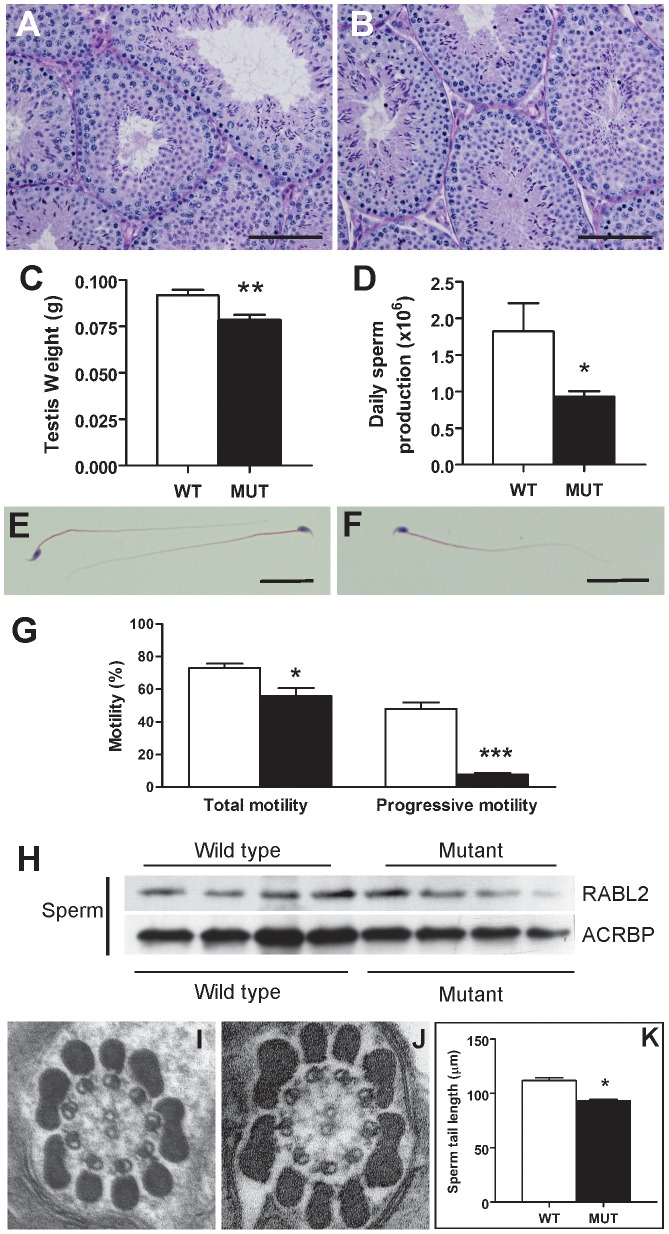
The sterility phenotype observed in *Rabl2^Mot/Mot^* males. Testis histology from a 10 weeks old wild type (A) and *Rabl2^Mot/Mot^* (B) male. Sections were stained with PAS. Spermatogenesis appeared qualitatively normal. Scale bars represent 100 µm. (C) Testis weights were significantly reduced in *Rabl2^Mot/Mot^* (Mut) animals compared to wild type (WT) littermates. (D) Daily sperm output from 10 weeks old wild type (WT) and *Rabl2^Mot/Mot^* (Mut) males. Haematoxylin and eosin stained sperm from wild type (WT) (E) and *Rabl2^Mot/Mot^* (Mut) (F) animals revealed no obvious differences between genotypes. Scale bars represent 25 µm (G) Sperm from 9 weeks old *Rabl2^Mot/Mot^* (black) however, had a significantly compromised ability for any form of motility and a very pronounced defect in the capacity for progressive motility compared to sperm from wild type (white) littermates. (H) Sperm from four different wild type and three different *Rabl2^Mot/Mot^* males (one mouse per lane) probed for RABL2 protein content. Protein loading was normalized using the sperm head protein ACRBP. (J–K) Electron microscopy of a cross-section of the mid-piece of a sperm from a wild type (WT) (I) and *Rabl2^Mot/Mot^* male (J) revealed no discernable difference in ultrastructure. (I) The length of sperm tails from wild type (WT) and *Rabl2^Mot/Mot^* (Mut) males. (* p<0.05, ** p<0.01, *** p<0.001).

All *Rabl2^Mot/Mot^* males examined were sterile when paired with wild type females (n>20 for periods of up to 6 months). With the exception of uniform male sterility, *Rabl2^Mot/Mot^* males were outwardly healthy, had normal body weights (Figure S1) and displayed normal mating behaviour up until at least 10 weeks of age when tissues were harvested for analysis. In order to define the cellular cause of male sterility, mice were phenotyped using the strategy outlined in Borg et al. [10]. Testes from *Rabl2^Mot/Mot^* males contained all germ cell types (Figure 3A–3B), however, testes weights were reduced by 15% compared to wild type littermates (91.8 mg versus 78.3 mg, p = 0.0094, n = 6 per group) (Figure 3C). An analysis of daily sperm production, as indicated by the number of Triton X-100-resistant nuclei, revealed output was reduced by 49% in *Rabl2^Mot/Mot^* males (1.82×10^6^ versus 0.93×10^6^, p = 0.046, n = 6) (Figure 3D). Collectively, these data indicate that germ cells were being lost during the latter part of spermatogenesis wherein they contribute relatively little to the overall testis weight.

Significant numbers of spermatozoa did reach the epididymis in *Rabl2^Mot/Mot^* males, and were thus available for ejaculation and fertilization (Figure S1B–S1C). Sperm structure appeared superficially normal (Figure 3E versus Figure 3F). Similarly, electron microscopy revealed no obvious structural abnormalities in either the axoneme and accessory structures (outer dense fibers, fibrous sheath and mitochondrial sheath) (Figure 3I–3J, n = 2 different genotype animal, 10 sperm per animal). Computer assisted sperm analysis of sperm from *Rabl2^Mot/Mot^* males, however, revealed low total motility (70.3% versus 51.8%, p = 0.0114, n = 6 per genotype) and minimal progressive motility (44.1% versus 7.4%, p = 0.000002, n = 6 per genotype) (Figure 3G). These data strongly suggest that *Rabl2^Mot/Mot^* males were sterile as a consequence of an inability of sperm to ascend the female reproductive tract following mating.

### RABL2 is evolutionarily conserved

Sequence analysis identified *Rabl2* as an uncharacterized member of the poorly characterized Rab-like clade of the Ras GTPase superfamily [11]. As indicated in Figure S2, RABL2 contains significant sequence similarity, and a likely evolutionary origin, to members of the RAB family, however, it forms a distinct branch within the superfamily – hence the name ‘RAB-like’. RABL proteins are defined by being closely related to, but excluded from the Rab clade due to the absence of one or more subfamily specific factors [12]–[14]. In particular, the predicted RABL2 protein from multiple organisms lacks the canonical C-terminal prenylation signal, is not identified as a Rab protein by Rabifier [14] and is excluded from the Rab clade on phylogenetic analysis using a selected training set of Rab sequences that encompass the diversity of Rab proteins in eukaryotes [15].

Although the precise biochemistry remains to be defined, recent data has suggested a role for two RABL proteins in cilia/flagella development. In particular, inactivation of *Rabl5* in *Trypanosoma brucei* resulted in stunted and immotile flagella [6], and IFT27 (RABL4) is a core component of the IFT (intra-flagellar transport) complex B machinery with a role in the anterograde delivery of proteins from the growing flagella tip in *Chlamydomonas* and RABL4 is also found in mammals [16]–[18]. In common with other members of the complex B, *Ift27* RNA interference results in stunted cilia [7].

A *RABL2* gene is visible within the human genome on chromosome 22q13.33 (called *RABL2B*) [19] (88.7% protein identity) and orthologues are present in many species, including the flagellated green algae *Chlamydomonas* (49% identity) and *Trypanosoma* species (51% identity) (Figure 1B). In *Homo sapiens* we note that *RABL2* has a paralogues expansion (*RABL2A*). With only four amino acid changes between the paralogues the functional significance is unclear. The notable exception to the existence of RABL2 orthologues in species containing cilia is in *Drosophila*, where an orthologue was not identified. The absence of *Rabl2* orthologues in organisms lacking cilia/flagella, as defined through a reverse BLAST search, is suggestive of an evolutionarily conserved role for RABL2 in cilia/flagella function, and is highly similar to the phylogenetic distribution of the *bona fide* IFT factor IFT22/RABL5 [6].

RT-PCR for *RABL2A* and *RABL2B* using whole human testis mRNA indicated that both paralogues are expressed in the testis (data not shown, MKOB). EST expression data suggests that RABL2A is expressed in a wide range of tissues including the brain, uterus, testis, lung, eye and prostate as well as a range of cancerous tissues (Unigene entry Hs.446425). EST expression data suggests that *RABL2B* is predominantly expressed in the brain, testis and uterus with lesser amounts in a range of other tissues (Unigene entry Hs.584862).

Of note, aspartic acid 73 is conserved in all likely orthologues in all species examined and is thus suggestive of it having a critical role in RABL2 function (Figure 1B). Western blotting of extract from equal number of sperm *Rabl2^WT/WT^* versus *Rabl2^Mot/Mot^* males revealed comparable levels of protein, indicating the phenotype was unlikely to be due to mRNA or protein instability, but rather the specific amino acid change (Figure 3H). These data also demonstrate that the D73 mutation does not impede RABL2 entry into the flagella/cilia compartment. This conclusion is also supported by the immunofluorescent localization of RABL2 in wild type versus mutant testis tissue sections (consideration should be given to the decreased numbers of elongated spermatids in mutant animals) (Figure 2D).

In common with the RAB GTPases, RABL2 possess all five consensus motifs required for GTP binding (Figure 1C), but it lacks the C-terminal prenylation signal required for membrane interactions classically associated with RAB function [20]. The possession of motifs involved in GTP/GDP binding in RAB proteins (Figure 1C) raised the possibility that RABL2 may also cycle between a GTP-bound ‘active’ form and a GDP-bound ‘inactive’ form.

In order to predict the effect of the Mot mutation on RABL2 function, and thus the underlying biochemical cause of the observed male sterility, the RABL2 protein sequence was aligned with the structure of multiple related Ras GTPases. Analyses revealed that the Mot mutation occurred in a RABL-specific amino acid in the center of a β-sheet critically involved in mediating a range of protein-protein interactions for the entire superfamily (for example RAB1B in complex with the guanidine dissociation factor and guanidine exchange factor, GEF, DRRA: pdb identifier 3JZA (Figure S3). In the context of RAB proteins, GEF proteins function to facilitate the exchange of GDP for GTP in the nucleotide binding site and thus, convert RABs from an inactive state to an active state wherein they are capable of binding to a specific set of effector proteins. If the same biochemistry is maintained in the RABL sub-group, we hypothesize that the Mot mutation would impede RABL2 binding to partner proteins, including with its GEF, and lead to the decreased conversion of inactive GDP-bound RABL2 into active GTP-bound RABL2. Ultimately, this would lead to the reduced delivery of effector proteins to target locations, in this instance the sperm tail.

### RABL2 binds to components of the IFT complex B, and GTP-bound RABL2 is essential for the delivery of key components to the developing sperm tail

**Figure 4:**
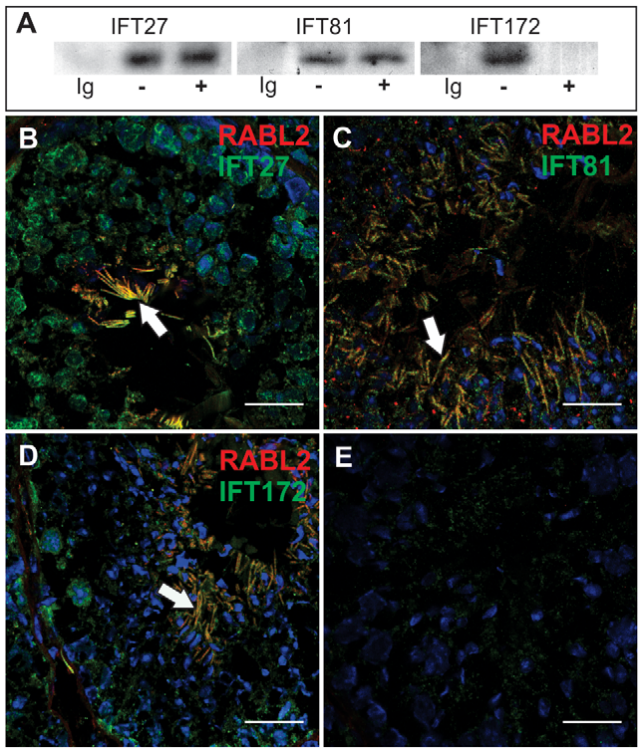
RABL2 binds to components of IFT complex B. (A) Immunoprecipitation of IFT complex B components (IFT27, IFT81 and IFT172) from testis extracts showed that all three were bound to RABL2. Ig = an immunoglobulin isotype and concentration matched control. −/+ indicate the presence of exogenous GTP in the testis homogenate to explore the potential regulation of binding to RABL2 by GTP binding status. (B–E) The co-localization of RABL2 (red) in the mid-piece of elongating spermatids (white arrows) within the seminiferous epithelium with each of IFT27 (B), IFT81 (C) and IFT172 (D). (E) A representative primary antibody control wherein the primary antibody was omitted from the staining protocol. Scale bars equal 25 µm.

The sequence homology between RABL2 and RABL4 in *Chlamydomonas* and RABL5 in *Trypanosomes* and the sperm tail phenotype in homozygous Mot mutant males, raised the possibility that RABL2 may also interact with components of the IFT machinery and have a role in defining sperm tail (motile cilia) length. In order to investigate this hypothesis, we perform immunoprecipitation using testis homogenate with specific IFT complex B component antibodies and then probed for RABL2 using Western blotting. Data revealed an interaction between RABL2 and all of IFT27, IFT81 and IFT172 (Figure 4A). Of interest, binding to IFT27 and IFT81 was apparently equal in the presence of GTP or GDP. IFT172 however, preferentially bound to GDP-RABL2. The significance of preferential binding to inactive RABL2 is currently unknown.

Immunofluorescent microscopy revealed the co-localization of RABL2 and each of IFT27, IFT81 and IFT172 within the mid-piece of elongated spermatids within the testis (Figure 4B–4E). Free (not co-localized with RABL2) IFT proteins were frequently observed within the principal piece of developing sperm, thus, raising the possibility that the annulus which sits at the junction between the mid- and principal-pieces may act as a barrier to RABL2 movement.

A role for RABL2 in defining sperm tail length was further supported by a 17% reduction in the length of sperm tails from *Rabl2^Mot/Mot^* males compared to those from wild type males (93 µm versus 111 µm, p<0.0001, n = 4 per genotype) (Figure 3K). Collectively, these data support a role for RABL2 in the assembly of the sperm tail. At present however, it is not possible to determine whether RABL2 is specifically involved in protein transport along the entire length of the cilia compartment, or if the sperm tail phenotype is secondary to a more generalized defect in RABL2-mediated (in conjunction with IFT components) protein transport in the cytosol (including the mid-piece of the sperm).

**Figure 5:**
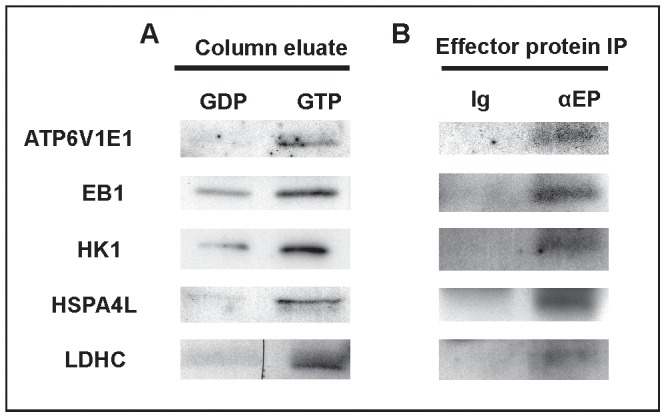
The identification of RABL2 effector proteins. (A) Preferential binding of candidate effector proteins to GTP-RABL2 (active state) over GDP (inactive state) was confirmed by Western blotting of additional affinity column eluates. (B) Specific binding of effector proteins to RABL2 was confirmed by immunoprecipitation of effector proteins (EP) from testis homogenates then probing for binding to RABL2 in Western blots. The specificity of immunoprecipitations was confirmed using parallel reactions wherein the precipitating antibody was replaced by isotype and concentration matched immunoglobulin.

As indicated above, the presence of the 5 motifs characteristic of GTP binding in RABs raises the possibility that RABL2 may cycle between a GTP-bound active state and a GDP-bound inactive state. If true, active GTP-bound RABL2 would be expected to bind to a set of effector proteins and deliver them to the developing sperm tail compartment, as the major site of RABL2 localization and the mediator of the *Rabl2^Mot/Mot^* phenotype. In order to test this hypothesis and identify putative effector proteins, recombinant RABL2 (isoform 2) was produced in *E.coli* and conjugated to agarose in either a GTP-bound or GDP-bound state and incubated with adult mouse testes extracts. Following extensive washing, protein bands preferentially bound to ‘active’ GTP-bound RABL2 were identified using mass spectrometry (Figure S4). 89 proteins were identified (Table S1). Of these, five were chosen for further analysis based on known roles in fertility or cilia function, the availability of analytical reagents and confirmation of preferential binding to the GTP-bound form of RABL2 (Figure 5A). Putative effector proteins analyzed included: ATP6V1E1 which is a protein exchanger localized to several ciliated tissues including the olfactoary epithelium [21]; the microtubule plus end trafficking protein EB1 which is involved in centrosome function and primary cilia development in the retina [22]; HK1 which is a component of the fibrous sheath of the sperm tail with a role in glycolysis [23]; the chaperone HSP4AL which has a role in prophase I of meiosis and haploid germ cell development [24]; and LDHC which is also a component of the glycolytic pathway and localized to the fibrous sheath [25]. Of note, genetic or chemical inhibition of LDHC or HK1 results in reduced ATP generation and the inhibition of axoneme microtubule sliding and thus, sperm immotility [23], [25]. Specific interactions between RABL2 and all five effector proteins were confirmed by co-immunoprecipitation using antibodies directed against the effector proteins (Figure 5B). These data indicate that in common with RAB proteins, GTP-bound RABL2 binds to a specific set of cargo proteins.

**Figure 6:**
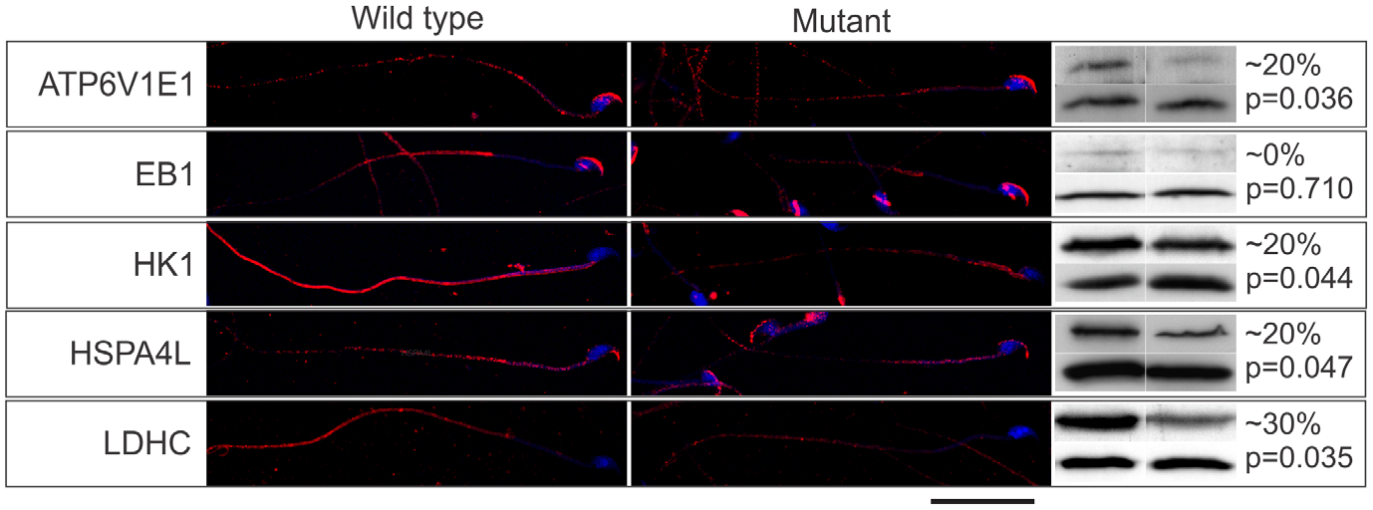
The Mot mutation resulted in the decreased delivery of effector proteins into sperm tails. The immunolocalization of RABL2 effector proteins (red) in sperm from wild type and *Rabl2^Mot/Mot^* mice. DNA was labelled with DAPI. The percentage change in total sperm content of individual effector proteins was quantitated using Western blotting blotting (right hand panel, upper bands). Sperm protein loading was normalized to sperm head protein ACRBP (right hand panel, lower bands). Each Western blot was done a total of three times and results averaged. This figure contains representative images. The approximate percentage change (rounded to the nearest 10% to reflect the non-linearity of ECL detection methods) and the p values are indicated. N = 3 animals per genotype per effector protein.

The effect of the Mot mutation on sperm development and the ultimate fate of the effector proteins was then tested through an examination of the relative effector proteins content, using immunofluorescence and Western blotting, on sperm collected from wild type and *Rabl2^Mot/Mot^* mice (n = 3 mice per genotype). Of note, sperm from wild type and *Rabl2^Mot/Mot^* mice were immunolabelled and photographed in parallel and under identical conditions. Within sperm from wild type mice ATP6V1E1, EB1, HK1 and LDHC were localized primarily to the principal piece of the sperm tail (Figure 6). Less intense staining for HK1 was observed within the mid-piece of the tail. HSPA4L staining was observed as speckled staining along both the mid- and principal pieces of the sperm tail. In addition, all of ATP6V1E1, EB1 and HSPA4L were observed in the peri-acrosomal region of the sperm head (Figure 6). Consistent with the hypothesis that the Mot mutation would lead to decreased delivery of effector proteins, sperm tails from *Rabl2^Mot/Mot^* mice contained relatively lesser amounts of all of ATP6V1E1, EB1, HK1s, HSP4AL and LDHC than sperm from wild type mice (Figure 6). The preferential localization of RABL2 to the mid-piece and of the effector proteins to the principal piece of the sperm tail under normal conditions and the residual localization of HK1 to the mid-piece, but not the principal piece, in sperm from *Rabl2^Mot/Mot^* males is suggestive of a role for RABL2 in the delivery of effector proteins up to the annulus, but not beyond. By contrast to the apparent decreased content of ATP61E1, EB1 and HSPA4L in the sperm tail, the association with the peri-acrosomal region of the sperm head was not obviously changed. This data is consistent with IFT transport being a specific requirement for protein transport into the cilia/flagella compartment.

Decreased effector protein content in sperm from *Rabl2^Mot/Mot^* males compared to sperm from wild types was confirmed by Western blotting (Figure 6). As a consequence of the apparent specific requirement of RABL2 for tail development, the relative effector protein content per sperm as measured by Western blotting needs to be qualified as it measured total sperm content i.e. head and tail. Regardless, all effector proteins, with the exception of EB1, were reduced.

Collectively, these data demonstrate that RABL2 function is regulated by GTP binding and that the D73 mutation compromised effector protein delivery into the growing sperm tail. The presence of residual levels of effector within the *Rabl2^Mot/Mot^* sperm suggest that the Mot mutation is either hypomorphic or some functional redundancy exists with other proteins.

### RABL2 shows an expression bias towards tissues containing motile cilia in the mouse

In order to define the distribution of RABL2 in the mouse, and thus tissues wherein additional pathology may be anticipated, a tissue survey for *Rabl2* expression was undertaken using quantitative PCR methods. *Rabl2* isoform 1 and 2 mRNA are both enriched within the male germ line (Figure 2E–2F). Both forms were however, widely expressed and notably within other tissues containing motile cilia including the lung, trachea, brain, ovary and kidney. Isoform 3 was not detected in any tissues examined (data not shown).

### Concluding comments

Through the use of random mutagenesis we have identified RABL2 as an evolutionarily conserved protein with an essential role in male fertility. As far as we can discern, the Mot line is the first model of *Rabl2* dysfunction in any species. Here we have demonstrated that RABL2 binds, in a GTP-regulated manner, to a specific set of effector proteins including key proteins involved in cilia development and function and delivers them into the growing sperm tail. Herein we have also defined the first component of a pathway by which components of the fibrous sheath are transported into the tail. Further, analogous to a recent report on the function of RABL5 in *Chlamydomonas reinhardtii* [26], RABL2 binds components of the IFT complex B in mammals, and RABL2 dysfunction results in shortened sperm tails. It is the absence of the effector proteins that likely mediates the sterility observed within the Mot mouse line.

The reproductive phenotype observed in the Mot mouse lines is remarkably similar to that seen in a sub-group of infertile men. Such men would be classified as having oligoasthenospermia i.e. decreased sperm output and severe motility defects but normal sperm morphology, and would usually be investigated for primary ciliary dyskinesia (PCD) [1], [27]. PCD is usually a recessive syndrome occurring in 1 in 20,000–60,000 live births (Mendelian Inheritance in Man no. 232,650) and is most frequently characterized by recurrent lung disease (from childhood), sinusitis and male infertility in adults [5], [28]. More variably PCD is associated with hydrocephalus, laterality defects and polycystic kidney disease. Known causes of human infertility include mutations in the axoneme component genes *DNAI2*, *DNAH5*, *DNAH11*, *DNAAF2* and *LRRC50*, *RSPH4A* and *RSPH9* which contribute to the development of the central tubules of the axoneme, *TXNDC* which is a thioredoxin and the coiled coiled proteins encoded by *CCDC39* and *CCDC40* which are involved in the early stages of axoneme assembly [29], [30]. Collectively however, the underlying aetiology remains unknown in ∼60% of cases. Several additional candidate genes have been identified using mouse studies e.g. *Pacrg* [31], *Spef2* [32], *Cby* [33], and *Pcdp1* [34]. It is clear however, from both human and mouse studies that the composition of cilia (including motile cilia) varies subtly between tissues, thus leading to a spectrum of clinical presentations in ciliopathies (http://v3.ciliaproteome.org, [35]). Regardless, *Rabl2* expression data suggests that RABL2 may be a candidate primary ciliary dyskinesia gene.

## Methods

### Identification of the Mot line

Animal procedures were approved by the Australian National University and Monash University Animal Experimentation Ethics Committees and performed in accordance with Australian NHMRC Guidelines on Ethics in Animal Experimentation. Point mutant mice were generated on a C57BL/6 background and outbred to CBA for two generations before being inter-crossed as described previously [8]. In order to identify lines containing sterility causing mutations, eight G3 brother-sister pairs per line were co-housed and the presence of pups monitored. If no pups were observed following six weeks, mice were re-paired with wild type partners to determine the origin of the infertility. The presence of copulatory plugs was monitored as an indication of mating behaviour. Lines wherein male sterility was observed in a ratio of approximately one in four with apparently normal mating behaviour were analyzed further.

### Identification of the infertility causing mutation in the Mot line and genotyping

The sterility causing mutation was mapped using a SNP-based method. Genomic DNA from five affected males was hybridized onto Affymetrix 5K mouse SNP Chips at the Australian Genome Research Facility and sequences compared to wild type C57BL6 and CBA sequences. The linkage interval was narrowed using additional mice and SNPs (www.well.ox.ac.uk/mouse/INBREDS/). SNP typing was performed using the Amplifluor SNP Genotyping System (Chemicon) and plates read in a BMG Fluostar Optima fluorescent microplate reader. The sequence of testis expressed candidate genes was determined by sequencing all protein coding exons and ∼50 bp of flanking introns through the Australian Genome Research Facility.

Following the identification of the phenotype causing mutation, mice were genotyped using the Amplifluor system using a wild type-specific primer 5′-GAAGGTCGGAGTCAACGGATTACAGAGTTGTGTTCTTGTTGCAGA-3′, a mutant allele primer 5′-GAAGGTGACCAAGTTCATGCTAGAGTTGTGTTCTTGTTGCAGG-3′, an antisense primer, 5′-AGCCTTGTGGTAGTAGGAAGCA-3′ and Platinum *Taq* DNA Polymerase (Invitrogen): 1 cycle, 95°C, 4 min; 35 cycles, 95°C, 10 sec; 60°C, 20 sec: 72°C, 40 sec and a final extension at 72°C, 3 min.

### Infertility characterization

Mot infertility was classified using the regime outlined in [10]. Daily sperm outputs were determined using the Triton X-100 nuclear solubilization method as described previously [36]. Sperm motility was assessed using computer assisted sperm analysis (n = 6/genotype) [37] and ultra-structure using electron microscopy (n = 2/genotype, average 10 sperm/mouse) [38]. Cauda epididymal sperm tail length was measured (10 weeks old, n = 4/genotype) following staining with hematoxylin and eosin. 40 tails/mouse were measured using Imaging technology MetaMorph software (Molecular Devices).

### Orthology searches and sequence alignment

To identify RABL2 orthologues, the mouse RABL2 isoform 2 sequence was used as a query in organism specific BLAST searches against protein sequence databases at NCBI. The highest scoring hit was reverse BLASTed against the mouse predicted proteome. Only sequences where mouse RABL2 came up as the highest identify were considered as containing RABL2 orthologues. The species databases searched were *Homo sapiens, Macaca mulatta, Trichomonas viginalis, Tetrahymena thermophila, Anolis carolinesis, Monodelphis domestica, Ornithorhynchus anatinus, Danio rerio, Gallus gallus, Xenopus (Silurana) tropicalis, Leishmania major, Chlamydomonas reinhardtii, Amphimedon queenslandica, Saccoglossus kowalevskii, Cryptosporidium parvum, Theileria parva, Dictyostelium discoideum, Caenorhabitis elegans, Cryptococcus neoformans, Guillardia theta, Saccharomyces cerevisiae, Physcomitrella patens, Arabidopsis thaliana, Nasonia vitripennis, Plasmodium falciparum, Drosophilia melanogaster, Nematostella vectensis, Monosiga brevicollis, Thalassiosira pseudonana, Phytophthora ramorum, Naegleria gruberi, Toxoplasma gondii, Cyanidioschyzon merolae, Paramecium tetraurelia, Trypanosome cruzi, Trypanosome brucei* and *Eimeria* tenella.

A selection of RABL2 orthologues were aligned using ClustalW2 (http://www.ebi.ac.uk/Tools/msa/clustalw2/). *Mus musculus* RABL2 transcript 2 sequence (ENSMUST00000023294,CCDS49704) was aligned with eight other taxa sequences obtained from NCBI (http://www.ncbi.nlm.nih.gov/). Sequences included: *Homo sapiens* (NP_009013.1 and NP_001003789.1); *Gallus gallus* (XP_424473.1); *Xenopus (Silurana) tropicalis* (NP_001072451.1); *Danio rerio* (NP_001038428.1); *Anolis carolinensis* (XP_003229013.1); *Leishmania major* (XP_001685503.1); *Chlamydomonas reinhardtii* (XP_001697212.1); and *Trypanosome brucei* (XP_829561.1).

### Construction of a phylogenetic tree

Selected RABL2 orthologues were aligned using muscle against a master RAB dataset that contained representatives of all RAB subfamilies previously identified as present in the last eukaryotic common ancestor, together with representative Ran sequences as an outgroup [15], [39]. The alignment was edited in Mesquite to remove regions of high divergence, and a phylogenetic tree built using Mr Bayes v3.2 and RAxML v7.0.3 [40], [41]. Highly divergent sequences were deleted and a second round of analysis performed. These data confirm that RABL2 falls within the RAB-like GTPase grouping, and are monophyletic, and hence a distinct paralogous family.

### RABL2 expression

The relative expression of *Rabl2* during post-natal testis development (day 0–70) and other adult tissues was defined using quantitative PCR using Brilliant Fast SYBR Green QPCR Master Mix (Stratagene) in the Agilent Mx 3000P QPCR System: 95°C, 2 min; 95°C, 5 sec; 60°C, 20 sec; 95°C, 2 min; then 72°C, 20 sec, for 50 cycles. Primers were: *Rabl*2201 5′-TCGATTAGCTGTGGCTTACAAA-3′ and 3′-CCTGTAAAACCTCGTCCATGA-5′; *Rabl2202* 5′-CTGCACCTGGGTGACAGTAA-3′ and 3′-CATCTTGGGAAGGGAAACAA-5′. 18S expression was used as a reference in post-natal testis expression analysis. Differential expression was analyzed using the 2^ΔΔCT^ method [42]. N = 3 separate mice per age.

### Recombinant RABL2 production

Full-length *Rabl2* (isoform 2) was amplified from wild type testes cDNA using the primers: 5′-ATAACCCGGGGTTCTGCAGGGGACAGAAACAGGCA-3′ and 5′-ATCTCCATGGTTAGGAAGGAGATGGGCTCTTG-3′, then cloned into the *XmaI* and *Ncol* sites of pET32a (Novagen). Recombinant RABL2 was produced and the histidine tag removed as described previously [43].

N-terminal tagged GST-RABL2 was generated using the Gateway cloning system (Life Technologies) according to manufacturer's instruction using pDEST15 vector and primers *Rabl2-attB1*: 5′-GGGGACAAGTTTGTACAAAAAAGCAGGCTTAGAAAACCTGTATTTTCAGGGCGCAGGGGACAGAAACAGG-3′ and *Rabl2-attB2*: 5′-AGCCCATCTCCTTCCTAATACCCAGCTTTCTTGTACAAAGTGGTCCCC -3′ with *pDOR222* and *pDEST15*. Recombinant protein was produced as described above.

### RABL2 antibody production

RABL2 mouse monoclonal antibodies were generated against recombinant RABL2 isoform 2 at the Monash University Antibody Technology Facility. Clones were amplified and purified as described [44]. Immunoglobulin was purified from media using Protein G FF sepharose beads (GE Healthcare), dialyzed against PBS and the concentration determined using the DC assay (Biorad).

### Immunofluorescent microscopy

RABL2 and IFT protein immuno-localization was done on fixed frozen testis sections and sperm smear post-fixed in 4% paraformaldehyde. Primary antibodies included RABL2 (RG2–G8) 6 µg/ml, IFT27 4 µg/ml (Santa Cruz), IFT81 10 µg/ml (Santa Cruz), IFT172 6 µg/ml (Santa Cruz). Effector proteins were localized on 1∶1 methanol:acetone fixed sperm [45]. Primary antibodies included: HK1 0.4 µg/ml (Sigma), ATP6V1E1 4 µg/ml (Abcam), EB1 4 µg/ml (Santa Cruz), HSPA4L 4 µg/ml (Abcam), LDHC 0.5 µg/ml (Sigma). Secondary antibodies included: donkey anti-mouse Alexa Fluor 555, donkey anti-rabbit Alexa Fluor 488, Alexa Fluor 555 and donkey anti-goat Alexa 555 (Life Technologies). DNA was visualized using 1 µg/ml 4′,6-diamidino-2-phenylindole (DAPI). Images were taken with Nikon C1 Eclipse C1 plus 90i upright automated microscope or Leica SP5 5 channels confocal invert microscope in the Monash University Microimaging Facility. Different excitation lasers (408 nm, 488 nm or 561 nm) were used depending on Alexa fluor dye conjugated to secondary antibody. The specificity of immunolabelling was determined by staining parallel sections in the absence of the primary antibody. The specificity of RABL2 staining was determined by pre-absorbing the antibody with a 500-fold molar excess of the immunizing peptide prior to immunochemistry in parallel with a non-preabsorbed positive control.

### The identification of RABL2 effector proteins and Western blotting

Putative RABL2 testis effector proteins were identified using RABL2 affinity columns loaded with either GTP or GDP to create active and inactive RABL2 as described previously [46]. Eluted proteins were visualized on 12% SDS-PAGE gels using Coomassie brilliant blue. Proteins that preferentially bound to GTP-RABL2 were excised and sequenced by nano-LC ESI MS/MS at the Australian Proteomics Analysis Facility. Only proteins with two or more unique peptides matches with a Mascot scores of at least 40 were considered as putative effector proteins. Of the 89 non-redundant proteins identified, five were chosen for further analysis based on known roles in sperm or cilia function and the availability of analytical reagents. Preferential binding to GTP-RABL2 was confirmed by Western blotting on additional column eluates and by co-immunoprecipitation using antibodies directed against putative effector proteins. Briefly, 2 mg of adult mouse testis lysate was incubated with 4 µg of the target antibody: HK1 (Santa Cruz), ATP6V1E1 (Abcam), EB1 (Santa Cruz), HSPA4L (Abcam), LDHC (Abnova) supplemented with 100 µM GTPγS (Jena Bioscience). Interacting proteins were captured by protein G magnetic beads (Millipore) and eluted with 0.1 M glycine, pH 2.7. Protein binding to RABL2 was assessed by Western blotting. Negative control reactions included incubation with immunoglobulin of the appropriate species and at a matching concentration.

The relative content of RABL2 and effector proteins in sperm was determined using Western blotting of protein from 2×10^6^ sperm per lane from wild type versus mutant mice (n = 3, each lane containing sperm from one mouse). Samples were separated by 12% SDS-PAGE and transferred onto PVDF membrane. Membrane was blocked using 5% skim milk in PBS followed by incubation with the primary antibody overnight at 4°C. Samples were probed with the following antibodies separately. ATP6V1E1 4 µg/ml, EB1 0.4 µg/ml, HSPA4L 4 µg/ml, HK1 0.04 µg/ml, LDHC 2 µg/ml. Sample loading was normalized using pro-acrosomal binding protein (ACRBP) content and values were averaged over the three mice and compared between genotypes. A sperm head protein was used for normalization as the use of a tail protein e.g. actin, would have skewed the data as a consequence of short sperm tails being a component of the phenotype. Positive band intensity was measured and analyzed by ImageJ (http://imagej.nih.gov/). P values of <0.05 were considered as significant.

The ACRBP antibody was raised against the peptides CEMNELYDDSWRSQSTG and CLLRNQNRKMSRMR in goats as described previously [8], [47] and used at a dilution of 1 in 100,000.

The potential for RABL2 to interact with components of the IFT pathway was explored using co-immunoprecipitations with 1 mg of adult testis lysate incubated with 4 µg of IFT172, IFT81, IFT27 (Santa Cruz) antibodies as discussed above. Immunoprecipitated complexes were separated on 12% SDS-PAGE gels then probed for RABL2 (5 µg/ml) in a Western blot as described above.

### Statistics

Student's t-test was used to compare the means of two populations using Graphpad Prism 5.0. P values<0.05 was used to define statistical significance.

## Supporting Information

Figure S1 The effect of the *Rabl2^Mot/Mot^* mutation on body weight and epididymal histology. (A) The *Rabl2^Mot/Mot^* mutation had no discernable effect of body weight at 9 weeks of age, n = 8. (B–C) Epididymal histology did not differ noticeably between WT (B) and *Rabl2^Mot/Mot^* animals (Mut, C). Scale bars equal 100 µm.
(TIF)

Figure S2 Phylogeny of selected representatives of RAB subfamilies in comparison to likely RABL2 orthologues. Numbers on internodes refer to MrBayes posterior probability/PhyML bootstrap support values and the PhyML topology is shown. RABL2 species included are as follows: *Homo sapiens* (RABL2A and RABL2B*), Mus musculus*, *Trypanosoma brucei*, and *Chlamydomonas reinhardtii*. Remaining data are a subset of sequences used for Figure 3 of Elias et al [15]. The reconstruction demonstrates that the RABL2 sequences are excluded from the true RAB group, *albeit* with evidence of being closer to RABs than the outgroup Ran. In his reconstruction there is evidence for a close relationship with the RTW RAB-like GTPases, and suggesting that RABL2 and RTW are monophyletic. In a separate reconstruction where the RTW sequences were removed, RABL2 sequences were robustly reconstructed as monophyletic, and also eliminating long branch attraction artifact. Together with the absence of some canonical sequence features, these data are consistent with the annotation of RABL2 as RAB-like.
(TIF)

Figure S3 The position of the Mot mutation within a RAB protein structure. The structure of Rab1 (green) bound to a GEF-domain (3JZA [48], blue). The position of D93 (Q60 in Rab1) at the Rab1/GEF interface is shown as red spheres (Q60 is in the equivalent position to D73 in RABL2). This position is close to the interface formed by RAB proteins with other binding partners including SEC2p. The figure was produced using PYMOLSEC2 p.
(TIF)

Figure S4 SDS-PAGE size fractionation of eluates from GTP-RABL2 (GTP, active) and GDP-RABL2 (GDP, inactive) affinity columns. Lines indicate the position of gel slices analysed by mass spectrometry.
(TIF)

Table S1 Putative RABL2 effector proteins.
(DOC)
