# Supplementary figures and images for: RAB-Like 2 Has an Essential Role in Male Fertility, Sperm Intra-Flagellar Transport, and Tail Assembly
Source: PLoS Genet. 2012 Oct 4;8(10):e1002969. doi: 10.1371/journal.pgen.1002969 (PMC3464206; doi:10.1371/journal.pgen.1002969)

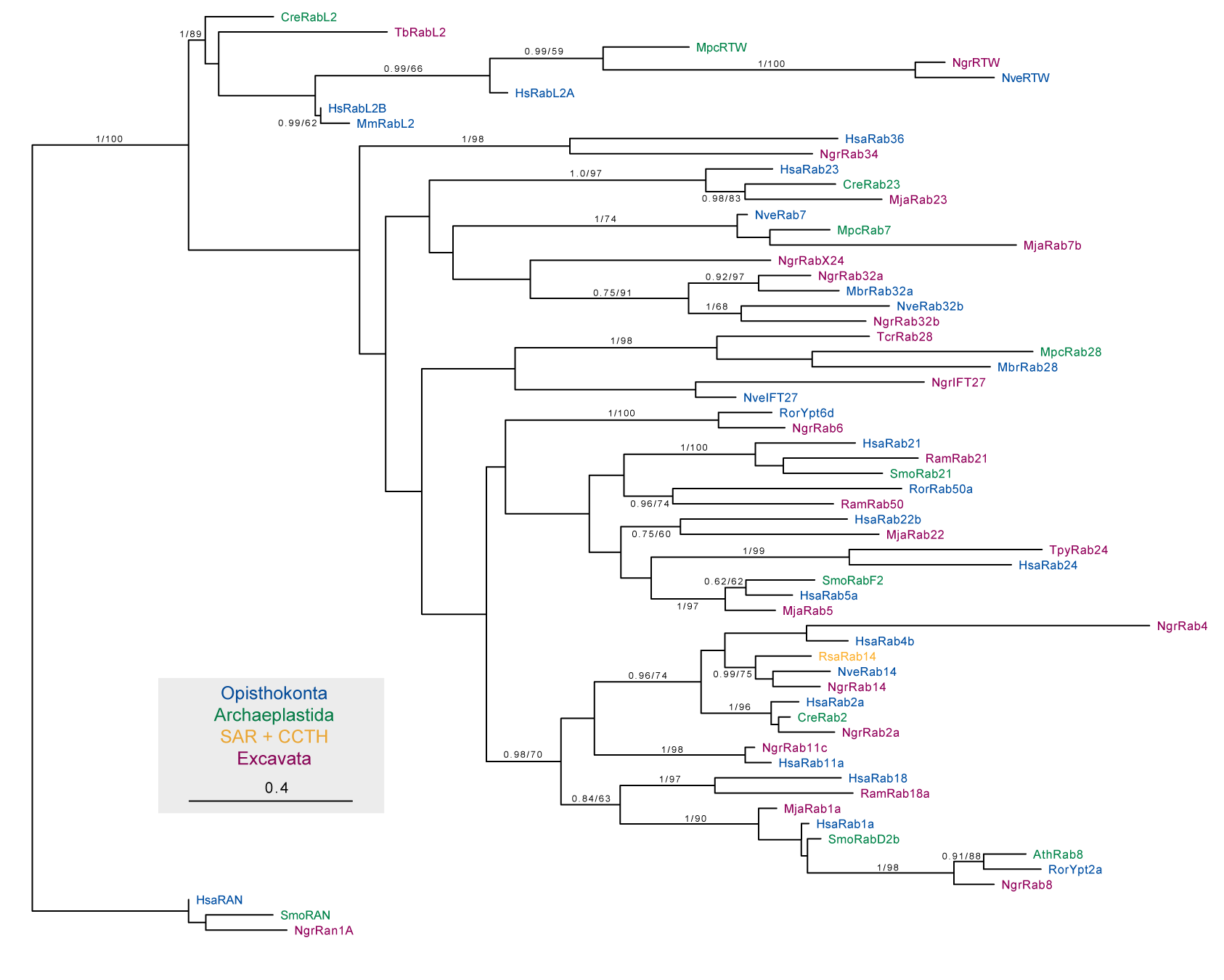

Supplement: Figure S2 — Phylogeny of selected representatives of RAB subfamilies in comparison to likely RABL2 orthologues. Numbers on internodes refer to MrBayes posterior probability/PhyML bootstrap support values and the PhyML topology is shown. RABL2 species included are as follows: Homo sapiens (RABL2A and RABL2B), Mus musculus, Trypanosoma brucei, and Chlamydomonas reinhardtii. Remaining data are a subset of sequences used for Figure 3 of Elias et al [15]. The reconstruction demonstrates that the RABL2 sequences are excluded from the true RAB group, albeit with evidence of being closer to RABs than the outgroup Ran. In his reconstruction there is evidence for a close relationship with the RTW RAB-like GTPases, and suggesting that RABL2 and RTW are monophyletic. In a separate reconstruction where the RTW sequences were removed, RABL2 sequences were robustly reconstructed as monophyletic, and also eliminating long branch attraction artifact. Together with the absence of some canonical sequence features, these data are consistent with the annotation of RABL2 as RAB-like. (TIF) [file pgen.1002969.s002.tif]

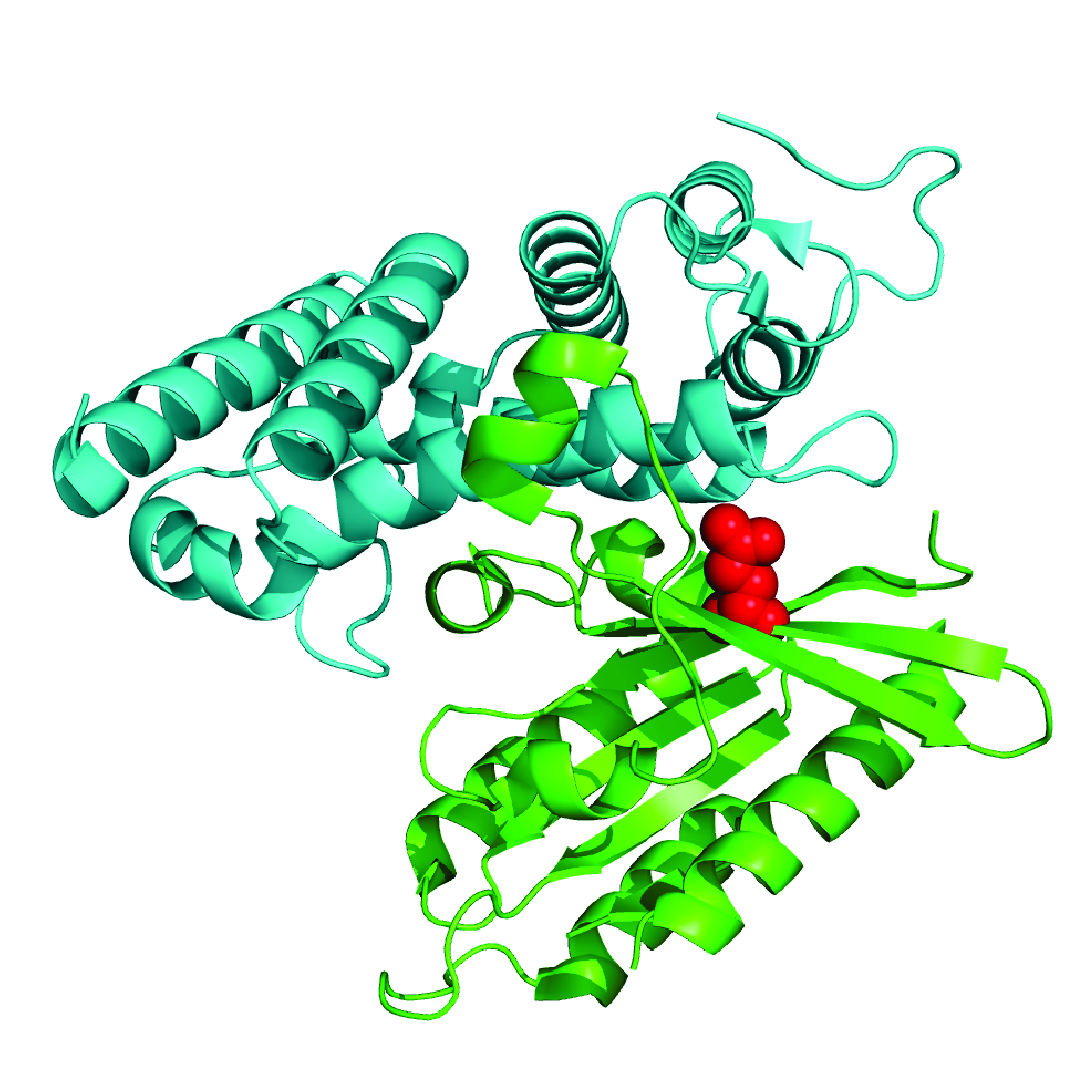

Supplement: Figure S3 — The position of the Mot mutation within a RAB protein structure. The structure of Rab1 (green) bound to a GEF-domain (3JZA [48], blue). The position of D93 (Q60 in Rab1) at the Rab1/GEF interface is shown as red spheres (Q60 is in the equivalent position to D73 in RABL2). This position is close to the interface formed by RAB proteins with other binding partners including SEC2p. The figure was produced using PYMOLSEC2 p. (TIF) [file pgen.1002969.s003.tif]

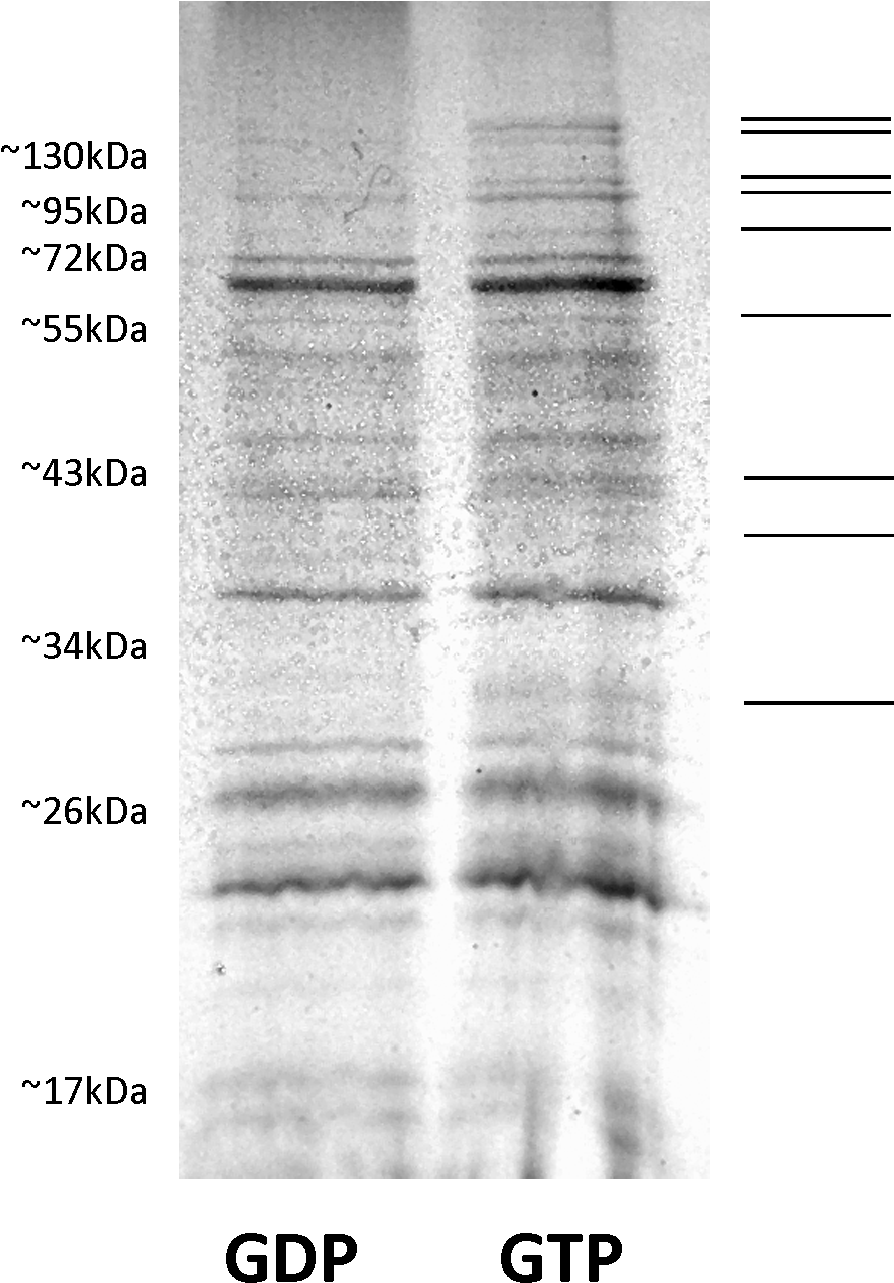

Supplement: Figure S4 — SDS-PAGE size fractionation of eluates from GTP-RABL2 (GTP, active) and GDP-RABL2 (GDP, inactive) affinity columns. Lines indicate the position of gel slices analysed by mass spectrometry. (TIF) [file pgen.1002969.s004.tif]
