# Supplementary material for: RAB-Like 2 Has an Essential Role in Male Fertility, Sperm Intra-Flagellar Transport, and Tail Assembly
Source: PLoS Genet. 2012 Oct 4;8(10):e1002969. doi: 10.1371/journal.pgen.1002969 (PMC3464206; doi:10.1371/journal.pgen.1002969)
Supplement: Table S1 — Putative RABL2 effector proteins. (DOC) [file pgen.1002969.s005.doc]

**Supplementary table 1: Putative RABL2 effector proteins**

| **Accession number** | **Protein name** | **MW** |
| --- | --- | --- |
| 1433E_MOUSE | 14-3-3 protein epsilon | 29155 |
| 2AAA_MOUSE | Serine/threonine-protein phosphatase 2A 65 kDa regulatory subunit A alpha isoform | 65281 |
| 4F2_MOUSE | 4F2 cell-surface antigen heavy chain | 58300 |
| ACE_MOUSE | Angiotensin-converting enzyme | 150822 |
| ACTA_MOUSE | Actin, aortic smooth muscle | 41982 |
| ACTN4_MOUSE | Alpha-actinin-4 | 104911 |
| AEDO_MOUSE | 2-aminoethanethiol dioxygenase | 28354 |
| APOA1_MOUSE | Apolipoprotein A-I | 30569 |
| APOE_MOUSE | Apolipoprotein E | 35844 |
| CA014_MOUSE | Uncharacterized protein C1orf14 homolog | 70903 |
| CAND1_MOUSE | Cullin-associated NEDD8-dissociated protein 1 | 136245 |
| CC104_MOUSE | Coiled-coil domain-containing protein 104 | 39575 |
| CLUS_MOUSE | Clusterin | 51623 |
| CTL2_MOUSE | Choline transporter-like protein 2 | 79871 |
| CUL3_MOUSE | Cullin-3 | 88891 |
| CUL4A_MOUSE | Cullin-4A | 87697 |
| CUL5_MOUSE | Cullin-5 | 90916 |
| DHAK_MOUSE | Bifunctional ATP-dependent dihydroxyacetone kinase/FAD-AMP lyase | 59653 |
| DLGP4_MOUSE | Disks large-associated protein 4 | 107970 |
| DPEP3_MOUSE | Dipeptidase 3 | 54213 |
| EF1B_MOUSE | Elongation factor 1-beta | 24678 |
| EF2_MOUSE | Elongation factor 2 | 95253 |
| EFHD2_MOUSE | EF-hand domain-containing protein D2 | 26775 |
| ENPL_MOUSE | Endoplasmin | 92418 |
| ENTP2_MOUSE | Ectonucleoside triphosphate diphosphohydrolase 2 | 54285 |
| ERP44_MOUSE | Endoplasmic reticulum resident protein 44 | 46823 |
| FA49B_MOUSE | Protein FAM49B | 36753 |
| FLNA_MOUSE | Filamin-A | 281018 |
| FTHFD_MOUSE | 10-formyltetrahydrofolate dehydrogenase | 98647 |
| GELS_MOUSE | Gelsolin | 85888 |
| GRP78_MOUSE | 78 kDa glucose-regulated protein | 72377 |
| HS74L_MOUSE | Heat shock 70 kDa protein 4L (HSPA4L) | 94322 |
| HS90A_MOUSE | Heat shock protein HSP 90-alpha | 84735 |
| HS90B_MOUSE | Heat shock protein HSP 90-beta | 83273 |
| HSP72_MOUSE | Heat shock-related 70 kDa protein 2 | 69698 |
| HSP74_MOUSE | Heat shock 70 kDa protein 4 | 94073 |
| HSP7C_MOUSE | Heat shock cognate 71 kDa protein | 70827 |
| HXK1_MOUSE | Hexokinase-1 (HK1) | 108233 |
| IMB1_MOUSE | Importin subunit beta-1 | 97090 |
| IPO5_MOUSE | Importin-5 | 123511 |
| IPO7_MOUSE | Importin-7 | 119410 |
| KCRB_MOUSE | Creatine kinase B-type | 42686 |
| LDHC_MOUSE | L-lactate dehydrogenase C chain (LDHC) | 35889 |
| LKHA4_MOUSE | Leukotriene A-4 hydrolase | 68977 |
| MAOX_MOUSE | NADP-dependent malic enzyme | 63958 |
| MARE1_MOUSE | Microtubule-associated protein RP/EB family member 1 (EB1) | 29997 |
| MEP50_MOUSE | Methylosome protein 50 | 36919 |
| NASP_MOUSE | Nuclear autoantigenic sperm protein | 83903 |
| NB5R3_MOUSE | NADH-cytochrome b5 reductase 3 | 34106 |
| NSF1C_MOUSE | NSFL1 cofactor p47 | 40685 |
| NUDC_MOUSE | Nuclear migration protein nudC | 38334 |
| PAIP1_MOUSE | Polyadenylate-binding protein-interacting protein 1 | 45673 |
| PHLD_MOUSE | Phosphatidylinositol-glycan-specific phospholipase D | 93196 |
| PLCD1_MOUSE | 1-phosphatidylinositol-4,5-bisphosphate phosphodiesterase delta-1 | 85847 |
| PPBT_MOUSE | Alkaline phosphatase, tissue-nonspecific isozyme | 57419 |
| PPM1G_MOUSE | Protein phosphatase 1G | 58691 |
| PSD11_MOUSE | 26S proteasome non-ATPase regulatory subunit 11 | 47407 |
| PSMD1_MOUSE | 26S proteasome non-ATPase regulatory subunit 1 | 105663 |
| PSMD2_MOUSE | 26S proteasome non-ATPase regulatory subunit 2 | 100139 |
| PSMD3_MOUSE | 26S proteasome non-ATPase regulatory subunit 3 | 60661 |
| RADI_MOUSE | Radixin | 68558 |
| RRBP1_MOUSE | Ribosome-binding protein 1 | 172776 |
| SFPQ_MOUSE | Splicing factor, proline- and glutamine-rich | 75394 |
| SPA3K_MOUSE | Serine protease inhibitor A3K | 46850 |
| SPESP_MOUSE | Sperm equatorial segment protein 1 | 44674 |
| SPT20_MOUSE | Spermatogenesis-associated protein 20 | 88416 |
| STIP1_MOUSE | Stress-induced-phosphoprotein 1 | 62542 |
| SYAC_MOUSE | Alanyl-tRNA synthetase, cytoplasmic | 106841 |
| TADBP_MOUSE | TAR DNA-binding protein 43 | 44519 |
| TBCB_MOUSE | Tubulin-folding cofactor B | 27368 |
| TCPG_MOUSE | T-complex protein 1 subunit gamma | 60591 |
| TCPZ_MOUSE | T-complex protein 1 subunit zeta | 57968 |
| TERA_MOUSE | Transitional endoplasmic reticulum ATPase | 89266 |
| TNPO1_MOUSE | Transportin-1 | 102291 |
| TNPO2_MOUSE | Transportin-2 | 100391 |
| TOM34_MOUSE | Mitochondrial import receptor subunit TOM34 | 34257 |
| TPM3_MOUSE | Tropomyosin alpha-3 chain | 32843 |
| TRFE_MOUSE | Serotransferrin | 76674 |
| TRXR3_MOUSE | Thioredoxin reductase 3 | 76067 |
| TSNAX_MOUSE | Translin-associated protein X | 32906 |
| UBA1_MOUSE | Ubiquitin-like modifier-activating enzyme 1 | 117734 |
| UBP5_MOUSE | Ubiquitin carboxyl-terminal hydrolase 5 | 95772 |
| USO1_MOUSE | General vesicular transport factor p115 | 106917 |
| VATE1_MOUSE | V-type proton ATPase subunit E 1 (ATP6V1E1) | 26141 |
| VINC_MOUSE | Vinculin | 116644 |
| VPS35_MOUSE | Vacuolar protein sorting-associated protein 35 | 91655 |
| XPO1_MOUSE | Exportin-1 | 123013 |
| XPO2_MOUSE | Exportin-2 | 110382 |
| XPO7_MOUSE | Exportin-7 | 123731 |
